# Pre- to postdiagnosis leisure-time physical activity and prognosis in postmenopausal breast cancer survivors

**DOI:** 10.1186/s13058-019-1206-0

**Published:** 2019-11-07

**Authors:** Audrey Y. Jung, Sabine Behrens, Martina Schmidt, Kathrin Thoene, Nadia Obi, Anika Hüsing, Axel Benner, Karen Steindorf, Jenny Chang-Claude

**Affiliations:** 1 Division of Cancer Epidemiology, German Cancer Research Center (DKFZ), Heidelberg, Germany; 2 Division of Physical Activity, Prevention and Cancer, German Cancer Research Center (DKFZ) and National Center for Tumor Diseases (NCT), Heidelberg, Germany; 3 Cancer Epidemiology Group, University Cancer Center Hamburg (UCCH), University Medical Center Hamburg-Eppendorf, Hamburg, Germany; 4 Institute for Medical Biometry and Epidemiology, University Medical Center Hamburg-Eppendorf, Hamburg, Germany; 5 Division of Biostatistics, German Cancer Research Center (DKFZ), Heidelberg, Germany

**Keywords:** Breast cancer, Prognosis, Survival, Physical activity, Postmenopausal

## Abstract

**Background:**

Physical activity (PA) before and after breast cancer diagnosis has been reported to be associated with lower mortality. However, whether changes in the activity after diagnosis impact prognosis is unclear and has not received much attention. This study aimed to examine pre- to postdiagnosis leisure-time PA and breast cancer prognosis.

**Methods:**

We used data from the MARIE study, a prospective population-based patient cohort study of 3813 postmenopausal breast cancer patients, aged 50–74 at diagnosis, recruited from 2002 to 2005, re-interviewed in 2009, and followed up until June 2015. Prediagnosis PA was assessed at recruitment; postdiagnosis PA was assessed at re-interview in 2009. To examine pre- to postdiagnosis change in PA, women were categorized by pre- and postdiagnosis PA using a cut-off of 7.5 MET-h/week for meeting PA recommendations and combined into four groups: insufficiently active, increasingly active, decreasingly active, and sufficiently active. Cox regression models with delayed entry were used to assess associations between pre- to postdiagnosis patterns of PA and overall mortality (OM), breast cancer mortality (BCM), and recurrence-free survival (RFS). Additional analyses of pre- and postdiagnosis PA (no activity (reference), low activity, sufficient activity) with cancer outcomes, such as using a time-dependent model, were performed. In total, 2042 patients were included in the analyses.

**Results:**

There were 206 deaths (114 from breast cancer) after a median follow-up time of 6.0 years after the 2009 interview. Compared to insufficiently active women, increasingly active women were at lower risk of OM, BCM, and RFS (HR (95%CI) of 0.50 (0.31–0.82), 0.54 (0.30–1.00), 0.58 (0.40–0.84), respectively). In sufficiently active women, associations for OM (0.75 (0.48–1.15)), BCM (0.61 (0.33–1.13)), and RFS 0.80 (0.57–1.14)) were similar to increasingly active women but attenuated, and decreasingly active women were not at lower risk for OM (0.91 (0.61–1.36)), BCM (0.80 (0.45–1.42)), and RFS (1.04 (0.76–1.43)). In time-dependent analyses, sufficient activity vs. no activity was associated with better OM (0.73 (0.57–0.93)), BCM (0.64 (0.46–0.89)), and RFS (0.82 (0.68–0.99)). Low activity was not significantly associated with prognosis.

**Conclusion:**

Our data support benefits for breast cancer prognosis in being physically active pre- and postdiagnosis particularly for women who were insufficiently active prediagnosis.

## Background

Physical activity before a diagnosis of primary breast cancer has been shown to be beneficial for survival in postmenopausal women [1]. Specifically, higher prediagnosis physical activity has been reported to be generally consistently inversely associated with overall [1] and breast cancer-specific mortality [1]. Postdiagnosis physical activity in relation to survival has received less attention, but results also generally consistently suggest that it may be associated with better overall survival [1]. The association between higher physical activity and lower risk of mortality after breast cancer diagnosis might be explained by body fatness, sex hormones, growth factors, adipokines, immune function, and inflammation [2, 3].

Despite the recognized benefits of pre- and postdiagnosis physical activity, only few studies have examined whether changing physical activity levels following breast cancer diagnosis impacts survival, for example, if increasing postdiagnosis physical activity can help improve survival or if decreasing postdiagnosis physical activity can worsen survival. Some studies have found physical activity to decrease after breast cancer diagnosis [4], while others have not [5].

To date, change in physical activity from before breast cancer diagnosis to after breast cancer diagnosis and survival has been examined in three studies—two from the USA [6, 7] and one from Norway [5]. Total physical activity change with mortality was investigated in one of these studies [5], while recreational physical activity was investigated in the two US studies [6, 7]. An additional study examined the postdiagnosis physical activity change (post-treatment to 1-year post-treatment as well as meeting physical activity guidelines) on breast cancer prognosis [8]. On balance, findings from these four studies are mainly consistent with slight discrepancies which could, in part, be due to the differences in populations, assessment of physical activity and definitions of change, and follow-up time. Increasing recreational physical activity postdiagnosis was associated with lower overall mortality [7], while decreasing postdiagnosis physical activity on a quantitative [6] and an ordinal scale [5] was associated with higher overall mortality. In contrast, another study found that while neither quantitative changes in total physical activity nor moderate-vigorous recreational physical activity were associated with mortality, meeting the physical activity guidelines at both time points was associated with decreased overall mortality compared to women who did not meet the guidelines at both time points [8].

In light of the current evidence, there is still a pressing need to address changes in physical activity and long-term breast cancer survivorship, especially in non-US-based populations that have considerably different physical activity levels. With this in mind, we have investigated the associations between pre- to postdiagnosis leisure-time physical activity with prognosis in postmenopausal women who were diagnosed with first primary breast cancer in Germany.

## Methods

### Study population

We used the data from the Mammary Carcinoma Risk Factor Investigation (MARIE) study [9]. This is a prospective population-based patient cohort study conducted in two regions of Germany. Between 2002 and 2005, 3813 German-speaking breast cancer patients 50–74 years of age at diagnosis with an incident histologically confirmed invasive breast cancer (ICD-10 C50) (stages I to IV) or in situ tumour (D05) (stage 0) were recruited from participating clinics and cancer registries. To be eligible, patients additionally had to reside in one of the study regions and be physically and mentally capable of participating in an hour and a half long in-person interview. Patients were identified through frequent monitoring of hospital admissions, surgery schedules, and pathology records of all clinics serving these regions and also through the Hamburg Cancer Registry. Interviews were conducted by trained interviewers using standard questionnaires person to person at recruitment and by telephone in 2009 at follow-up for postdiagnosis physical activity and other lifestyle, personal, and clinical characteristics. Information on vital status was collected from population registries in 2009 [10] and 2015.

These studies were approved by the ethical committees of the University of Heidelberg, the Medical Board of the State of Rhineland-Palatinate and the ethical review board of Hamburg Medical Council, and were conducted in accordance with the Declaration of Helsinki. All study participants provided informed written consent.

**Fig. 1:**
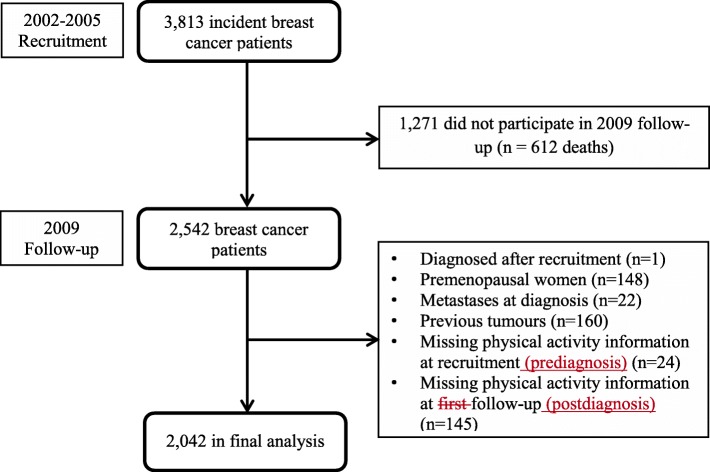
Flow diagram of the inclusion and exclusion criteria for participants of the MARIE study for analyses relating to the changes in physical activity levels and overall mortality, breast cancer mortality, and recurrence-free survival

For the present analysis, we considered women who completed both the recruitment and follow-up interviews (*n* = 2542) and used exposure information relating to physical activity, lifestyle, demographic, socio-economic, clinical, and other participant characteristics ascertained at these two time points. Women were excluded if they were recruited as a control at recruitment and later developed breast cancer during follow-up (*n* = 1), premenopausal (*n* = 148), had had metastases at diagnosis (*n* = 22), previous tumours other than breast cancer before diagnosis (*n* = 160), missing prediagnosis physical activity information (*n* = 24), and missing postdiagnosis physical activity information (*n* = 145), leaving 2042 women for the analyses (Fig. 1).

### Assessment of physical activity

At recruitment, physical activity was assessed during in-person interviews using a questionnaire that was designed and evaluated for participants in our study based on the existing validated questionnaires [11] and experiences from previous analyses of physical activity questionnaires [12]. At the follow-up, physical activity was assessed during telephone interviews using a questionnaire that was based on the recruitment questionnaire. Physical activity assessment included self-reported participation in walking and cycling for the purposes of commuting/transportation as well as recreational activities, sports, and fitness. Metabolic equivalent task-hours per week (MET-h/week) were calculated by multiplying the average hours per week spent on each activity with an individual intensity score [13]. Leisure-time physical activity is defined as additional activities related to recreational physical activities, sports, and fitness [14]. We have evaluated leisure-time physical activity and not total physical activity in relation to prognosis in this analysis. Total physical activity would also include walking and cycling for commuting/transportation in addition to other domains. In Germany, walking and cycling are usual methods for transportation. They are generally not performed with moderate intensity but with light intensity and so do not produce noticeable increases in breathing and heart rate [15]. For this reason, we focus on leisure-time physical activity.

#### Prediagnosis physical activity

Prediagnosis physical activity was determined from the information collected at the recruitment interview (median 3.8 months after diagnosis). Women were asked about their physical activity from the age of 50 until diagnosis. They were asked to list up to three leisure-time activities in which they most frequently participated. For each activity, they were asked to provide, from the age of 50 until diagnosis, the number of years, months per year, either days per week or days per month, and the number of hours/minutes per day they participated. Additionally, they were asked how much time (hours/minutes) they spent walking outside the home and cycling as a form of commuting or everyday cycling.

#### Postdiagnosis physical activity

Postdiagnosis physical activity was determined from information collected at the follow-up interview in 2009 (median 5.8 years after diagnosis) and was the physical activity performed from 3 months after diagnosis of breast cancer to the follow-up interview. Patients were asked if they had (re-)started any (other) leisure-time activities and to list up to four of these. For each activity, they were asked when they started (month/year), if they stopped (month/year) or if they were still participating, and the number of days per week and hours or minutes per day that they participated in each activity. They were additionally asked how much time (hours/minutes) they spent walking outside the home and cycling as a form of commuting or everyday cycling.

### Primary exposure of interest

#### Pre- to postdiagnosis change in leisure-time physical activity

A woman was termed insufficiently active if she did not achieve the minimum physical activity level recommended by the World Health Organization and Germany’s national guidelines, which are based on recommendations from the World Health Organization as well as other countries’ national guidelines—at least 150 min/week of moderate-intensity physical activity (equivalent to at least 7.5 MET-h/week). Conversely, a woman was classified as sufficiently active if she did achieve at least 7.5 MET-h/week [16, 17]. Four activity patterns were created to assess pre- to postdiagnosis physical activity: insufficiently active, increasingly active, decreasingly active, and sufficiently active. Categorization of these groups was based on the MET-hours/week values from leisure-time physical activity.

### Outcome assessment

Vital status was retrieved through central population registry databases of the study regions up to the end of June 2015, followed by requests for death certificates from local health offices. Cause of death was coded according to the 10th revision of the International Classification of Diseases (ICD-10-WHO). Second cancers, recurrences pertaining to the primary breast cancer, and metastatic events were ascertained from medical records or through contact with the treating physicians to verify information collected at the follow-up interviews. Primary study outcomes were overall mortality and breast cancer mortality, and secondary study outcome was recurrence-free survival. The event of interest in the overall mortality analyses was death attributed to any cause. The event of interest in breast cancer mortality analyses was death attributed to breast cancer (coded as ICD-10-C50), and deaths from other causes were censored at the date of occurrence. Events of interest in recurrence-free survival analyses were ipsilateral, local/regional invasive breast cancer recurrence, distant recurrence and metastases occurring after the primary diagnosis, and death [18]. Thus, recurrence-free survival is equivalent to risk for one of the mentioned events of interest. Participants without events of interest were censored at the date of last contact or 30 June 2015, whichever came first.

### Statistical analysis

The distribution of demographic, lifestyle, clinical, and tumour characteristics according to the four activity patterns was examined and compared using ANOVA.

The exposure of interest in our analysis was patterns for pre- to postdiagnosis leisure-time physical activity. Women who were insufficiently active served as the reference. Delayed-entry Cox proportional hazard models, based on the time since the follow-up interview in 2009 until the event of interest/censoring, were used to estimate hazard ratios (HRs) and corresponding 95% confidence intervals (CIs) for the associations between patterns for pre- to-postdiagnosis physical activity and overall and breast cancer mortality (primary outcomes) and recurrence-free survival (secondary outcome). The proportional hazards assumption was examined by visualizing the effect of a potential time-dependent covariate on the risk of outcome throughout the follow-up time, using a weighted least-squares line fitted to the residual plot as proposed by Grambsch et al. [19]. There was no violation of the proportional hazard assumption upon visual examination of potential time-dependent covariates on the risk of overall mortality, breast cancer mortality, and recurrence-free survival.

Besides investigating pre- to postdiagnosis leisure-time physical activity change using the recommendation as a cut-off, leisure-time physical activity was modelled as a time-dependent categorical variable (no activity (MET-h/week = 0) (reference), low activity (> 0 to < 7.5 MET-h/week), sufficient activity (≥ 7.5 MET-h/week)) using Cox proportional hazards models whereby the information for the variable at recruitment (prediagnosis) was updated at the time of follow-up (postdiagnosis) [20]. In addition, the associations between prediagnosis leisure-time physical activity as well as postdiagnosis leisure-time physical activity and cancer outcomes were investigated separately. In the time-dependent models as well when associations between prediagnosis leisure-time physical activity and prognosis were evaluated, the date of diagnosis was used as the starting time.

All models constructed to evaluate the associations between pre- to postdiagnosis change in leisure-time physical activity and the cancer outcomes were stratified (to allow for variation in baseline hazard) by study centre and age at diagnosis in 5-year categories. The Cox model included the prognostic factors tumour size (≤ 2 cm, 2–5 cm, > 5 cm, growth into chest wall, neoadjuvant chemotherapy, in situ), nodal status (0, 1–3, > 3, neoadjuvant chemotherapy, in situ), tumour grade (low/moderate, high), and ER/PR status (ER+/PR+, ER+/PR− or ER−/PR+, ER−/PR−, neoadjuvant chemotherapy, in situ), as well as mode of tumour detection (self-detected by palpation/secretion/pain, routine examination/mammography/ultrasound), menopausal hormone use at diagnosis (yes/no), recurrences between recruitment and follow-up (yes/no), time between recruitment and follow-up (continuous), and age at diagnosis (continuous). Other covariates tested and not included were determined a priori and did not change the risk estimates by at least 10%: BMI at the follow-up, smoking at the follow-up, education, HER2 status, type of surgery, chemotherapy, radiation therapy, tamoxifen/aromatase inhibitor therapy, and comorbidities (diabetes, cardiovascular disease, and osteoporosis). The time-dependent Cox models included the same covariates mentioned above with the exception of time between recruitment and follow-up, as the time-dependent model accounts for this inherently. The association analysis of postdiagnosis physical activity with outcomes was stratified by prediagnosis physical activity (insufficiently active/sufficiently active). The models constructed to investigate the prediagnosis associations with cancer outcomes were adjusted for tumour size, nodal status, grade, ER/PR status, mode of detection, menopausal hormone use at diagnosis, age at diagnosis, chemotherapy, and hormone therapy, and were stratified by study centre and age at diagnosis in 5-year categories. Categories of all variables can be seen in Table 1.

**Table 1:** Postdiagnosis characteristics of the population by change in recommended levels of leisure-time physical activity pre- and postdiagnosis

	Total	Pre- and postdiagnosis leisure-time physical activity			
		Insufficiently active^a^	Increasingly active	Decreasingly active	Sufficiently active
*n* (%)	2042 (100.0)	1037 (50.8)	348 (17.0)	285 (14.0)	372 (18.2)
Age at diagnosis, years, median (IQR)^b^	62.9 (58.6–66.4)	63.4 (59.1–67.1)	61.9 (58.0–65.3)	64.1 (59.5–67.1)	61.1 (57.3–64.4)
Physical activity prediagnosis, median (IQR)					
Walking (hours/week)^b^	6.0 (3.2–9.0)	6.0 (3.0–9.0)	6.0 (3.5–9.0)	7.0 (3.5–10.5)	6.0 (3.3–9.0)
Cycling (hours/week)^b^	1.2 (0.0–3.5)	1.0 (0.0–3.5)	1.4 (0.0–4.0)	1.8 (0.0–4.0)	2.0 (0.0–4.0)
Total (MET-h/week)^b^	43.1 (26.5–65.6)	35.0 (20.0–56.0)	38.7 (24.2–57.1)	59.2 (41.1–85.6)	59.5 (41.7–78.7)
Physical activity postdiagnosis, median (IQR)					
Walking (hours/week)^b^	6.0 (3.0–10.0)	6.0 (3.0–8.0)	6.0 (4.0–10.0)	6.0 (3.0–10.0)	7.0 (4.0–10.0)
Cycling (hours/week)^b^	1.0 (0.0–3.0)	0.0 (0.0–2.3)	2.0 (0.0–4.0)	1.0 (0.0–3.0)	2.0 (0.0–3.5)
Total (MET-h/week)^b^	40.0 (23.4–66.0)	29.6 (16.0–48.0)	56.6 (37.3–80.1)	33.1 (17.2–56.0)	62.2 (40.3–95.6)
BMI, median (IQR)^b^	25.3 (22.9–28.4)	25.8 (23.3–29.4)	25.0 (22.8–28.3)	24.8 (22.9–27.7)	24.4 (22.5–27.1)
Smoking status, *n* (%)					
Never	1108 (54.3)	580 (55.9)	179 (51.4)	157 (55.1)	192 (51.6)
Former	748 (36.6)	341 (32.9)	141 (40.5)	105 (36.8)	161 (43.3)
Current	186 (9.1)	116 (11.2)	28 (8.0)	23 (8.1)	19 (5.1)
Alcohol, g/day, median (IQR)^b^	0.7 (0.0–5.7)	0.6 (0.0–5.4)	0.7 (0.0–5.7)	2.0 (0.0–7.1)	1.4 (0.0–8.3)
Education, *n* (%)^b^					
Less than high/middle school	1141 (55.9)	659 (63.5)	182 (52.3)	151 (53.0)	149 (40.1)
High/middle school	583 (28.6)	268 (25.8)	108 (31.0)	79 (27.7)	128 (34.4)
College or university	318 (15.6)	110 (10.6)	58 (16.7)	55 (19.3)	95 (25.5)
Menopausal hormone use, *n* (%)	1000 (49.0)	483 (46.6)	171 (49.1)	150 (52.6)	196 (52.7)
Tumour size, cm, *n* (%)					
< 2	1159 (56.8)	566 (54.6)	199 (57.2)	168 (58.9)	226 (60.8)
2–5	622 (30.5)	336 (32.4)	101 (29.0)	86 (30.2)	99 (26.6)
> 5 and growth into chest wall/skin	73 (3.6)	46 (4.4)	11 (3.2)	8 (2.8)	8 (2.2)
Neoadjuvant chemotherapy^c^	61 (3.0)	32 (3.1)	11 (3.2)	6 (2.1)	12 (3.2)
In situ^3^	124 (6.1)	54 (5.2)	26 (7.5)	17 (6.0)	27 (7.3)
Number of positive lymph nodes, *n* (%)					
0	1322 (64.7)	690 (66.5)	209 (60.1)	170 (59.6)	253 (68.0)
1–3	400 (19.6)	183 (17.6)	72 (20.7)	79 (27.7)	66 (17.7)
> 3	135 (6.6)	78 (7.5)	30 (8.6)	13 (4.6)	14 (3.8)
Tumour grade, *n* (%)					
Low/moderate	1422 (69.6)	720 (69.4)	240 (69.0)	197 (69.1)	265 (71.2)
High	426 (20.9)	223 (21.5)	71 (20.4)	64 (22.5)	68 (18.3)
Hormone receptor status, *n* (%)					
ER+/PR+	1238 (60.6)	628 (60.6)	208 (59.8)	175 (61.4)	227 (61.0)
ER+/PR−, ER−/PR+	344 (16.8)	174 (16.8)	55 (15.8)	51 (17.9)	64 (17.2)
ER−/PR−	275 (13.5)	149 (14.4)	48 (13.8)	36 (12.6)	42 (11.3)
HER2 status, *n* (%)					
HER2+	312 (15.3)	159 (15.3)	63 (18.1)	46 (16.1)	44 (11.8)
HER2−	1413 (69.2)	715 (68.9)	228 (65.5)	197 (69.1)	273 (73.4)
Missing, *n* (%)	132 (6.5)	77 (7.4)	20 (5.7)	19 (6.7)	16 (4.3)
Type of surgery, *n* (%)					
Mastectomy	55 (2.7)	24 (2.3)	11 (3.2)	9 (3.2)	11 (3.0)
Mastectomy + axilla	478 (23.4)	266 (25.7)	85 (24.4)	57 (20.0)	70 (18.8)
Breast-conserving therapy	181 (8.9)	86 (8.3)	31 (8.9)	25 (8.8)	39 (10.5)
Breast-conserving therapy + axilla	1326 (64.9)	660 (63.6)	221 (63.5)	193 (67.7)	252 (67.7)
Mode of tumour detection, *n* (%)^b^					
Self-detected by palpation/secretion/pain	1007 (49.3)	550 (53.0)	151 (43.4)	136 (47.7)	170 (45.7)
Routine examination, mammography, ultrasound	1031 (50.5)	485 (46.8)	195 (56.0)	149 (52.3)	202 (54.3)
Chemotherapy, *n* (%)	917 (44.9)	481 (46.4)	163 (46.8)	127 (44.6)	146 (39.2)
Radiation therapy, *n* (%)	1627 (79.7)	828 (79.8)	274 (78.7)	230 (80.7)	295 (79.3)
Hormone therapy, *n* (%)	1651 (80.9)	842 (81.2)	276 (79.3)	228 (80.0)	305 (82.0)
Diabetes, *n* (%)^b^	239 (11.7)	162 (15.6)	21 (6.0)	32 (11.2)	24 (6.5)
Cardiovascular disease, *n* (%)^b^	1349 (66.1)	737 (71.1)	227 (65.2)	180 (63.2)	205 (55.1)
Osteoporosis, *n* (%)	452 (22.1)	241 (23.2)	74 (21.3)	69 (24.2)	68 (18.3)

^a^Insufficiently active is defined as having between 0 and 7.5 MET-h/week of leisure-time physical activity; sufficiently active is defined as having ≥ 7.5 MET-h/week of leisure-time physical activity

^b^Statistically significant differences in patient characteristics between physical activity groups tested by ANOVA (*p* < 0.05 for statistical significance)

^c^Also for number of positive lymph nodes, tumour grade, ER/PR status, and HER2 status

Possible effect modification of the associations between pre- to postdiagnosis physical activity and overall mortality by ER status, HER2 status, BMI, chemotherapy, radiation therapy, and smoking status was examined by applying the likelihood ratio test to a model with the interaction term of the main exposure and the potential modifier and to a model without the interaction term.

In sensitivity analyses, all analyses were repeated for all three outcomes excluding (1) women who developed a recurrence (ipsilateral, local/regional, distant and metastatic recurrence, or a second tumour) by the follow-up interview, (2) women with in situ tumours, and (3) women who did not walk for at least 10 min 3 months after the operation. In situ tumours have been reported to have a better prognosis than invasive tumours, and women who did not walk for at least 10 min may not be well enough to exercise. Complete case analysis was performed, as the proportion of missing was less than 1.7% for all variables except for HER2 (6.5% missing).

All tests of statistical significance were two-sided, and the significance level was set to 0.05. Analyses were conducted using the SAS statistical software package (version 9.4).

## Results

Median age at breast cancer diagnosis was 62.9 years. By 30 June 2015, a median of 6.0 years after the follow-up interview, 206 (10.1%) women died, 115 (5.6%) of which were from breast cancer, and 324 women (16.1%) developed a breast cancer recurrence (*n* = 132 of which occurred between the initial breast cancer diagnosis and the follow-up interview in 2009).

In our patient cohort, 1349 women (66.1%) participated in at least 1 leisure-time physical activity prediagnosis, and 1253 women (61.4%) participated in at least 1 leisure-time physical activity postdiagnosis. Callisthenics and swimming were the most reported activities both pre- and postdiagnosis. For women included in our analysis, median energy expenditure from leisure-time physical activity was 3.4 MET-h/week prediagnosis and 4.5 MET-h/week postdiagnosis. Based on the leisure-time physical activity at pre- and postdiagnosis, 657 and 720 women, respectively, met the physical activity recommendations of achieving at least 150 min of moderate-intensity physical activity.

There were differences between the pre- to postdiagnosis leisure-time physical activity groups with respect to age at diagnosis, walking, cycling, and total physical activity (walking and cycling for transportation in addition to leisure-time physical activity) at recruitment and follow-up, BMI, education, alcohol consumption, mode of tumour detection, cardiovascular disease, and diabetes (Table 1). At prediagnosis, walking levels were highest amongst women who had decreased activity, and cycling levels were higher amongst women who were active prediagnosis compared to women who were insufficiently active prediagnosis. At postdiagnosis, walking levels were highest amongst women who were sufficiently active, while cycling was highest amongst women who were active postdiagnosis. Adding walking or cycling as a covariate to the models estimating the associations between pre- and postdiagnosis leisure-time physical activity change with prognosis did not change the risk estimates, so neither walking nor cycling was included in the presented models.

In the analyses evaluating the changes in leisure-time physical activity, compared to women who were insufficiently active, women who were increasingly active had a significantly decreased risk for overall mortality (HR 0.50, 95% CI 0.31–0.82) (Table 2). There were suggestions for non-significantly decreased risk for overall mortality (HR 0.75, 95% CI 0.48–1.15) for women who were sufficiently active but not for women who were decreasingly active (HR 0.91, 95% CI 0.61–1.36).

**Table 2:** Associations between change in pre- and postdiagnosis leisure-time physical activity according to recommendations and overall mortality, breast cancer mortality, and recurrence-free survival in postmenopausal breast cancer survivors

Pre- to postdiagnosis leisure-time physical activity patterns	Number	Overall mortality		Breast cancer mortality		Recurrence-free survival	
		Events	HR (95% CI)	Events	HR (95% CI)	Events	HR (95% CI)
Insufficiently active	1021	128	1.00 (ref.)	71	1.00 (ref.)	190	1.00 (ref.)
Increasingly active	345	20	0.50 (0.31–0.82)	14	0.54 (0.30–1.00)	36	0.58 (0.40–0.84)
Decreasingly active	281	32	0.91 (0.61–1.36)	16	0.80 (0.45–1.42)	54	1.04 (0.76–1.43)
Sufficiently active	372	26	0.75 (0.48–1.15)	13	0.61 (0.33–1.13)	44	0.80 (0.57–1.14)

Analyses were adjusted for age at diagnosis, tumour size, nodal status, grade, ER/PR status, mode of detection, menopausal hormone use at diagnosis, recurrences between diagnosis and follow-up, and time between recruitment and follow-up, and were stratified by study centre and age at diagnosis in 5-year categories

Patterns for breast cancer mortality and recurrence-free survival were similar to those for overall mortality, where being increasingly active was associated with a decreased risk of breast cancer mortality (HR 0.54, 95% CI 0.30–1.00) and recurrence-free survival (0.58, 95% CI 0.40–0.84). Being sufficiently active also seemed to be non-significantly associated with decreased risk of breast cancer mortality (HR 0.61, 95% CI 0.33–1.13) and recurrence-free survival (HR 0.80, 95% CI 0.57–1.14). Being decreasingly active was not associated with mortality from breast cancer (HR 0.80, 95% CI 0.45–1.42) or with recurrence-free survival (HR 1.04, 95% CI 0.76–1.43).

There was no effect modification by ER status, HER2 status, BMI, chemotherapy, radiation therapy, or smoking status in the relationships between pre- to postdiagnosis leisure-time physical activity and overall survival (all *P* > 0.05). Additionally, there was no meaningful deviation in risk estimates from sensitivity analyses for all three outcomes when excluding (1) women who developed a recurrence before follow-up, (2) women with in situ tumours, and (3) women who did not sometimes walk for at least 10 min 3 months after the operation.

In time-dependent Cox models, compared to women who did no leisure-time physical activity, women who engaged in sufficient activity had decreased overall mortality (HR 0.73, 95% CI 0.57–0.93), breast cancer mortality (HR 0.64, 95% CI 0.46–0.89), and better recurrence-free survival (HR 0.82, 95% CI 0.68–0.99) (Table 3). There was no association between low activity and prognosis in the results from time-dependent Cox models. Prediagnosis physical activity was also not associated with long-term breast cancer prognosis. On the other hand, in a subgroup of women who were insufficiently active prediagnosis, those sufficiently active postdiagnosis compared to no activity postdiagnosis had a decreased risk of overall mortality (HR 0.43, 95% CI 0.26–0.72), breast cancer mortality (HR 0.48, 95% CI 0.25–0.91), and better recurrence-free survival (HR 0.59, 95% CI 0.40–0.86). In a subgroup of women who were sufficiently active prediagnosis, low activity postdiagnosis compared to no activity postdiagnosis was associated with decreased risk of overall mortality (HR 0.38, 95% CI 0.16–0.88).

**Table 3:** Time-dependent associations between leisure-time physical activity and overall mortality, breast cancer mortality, and recurrence-free survival in postmenopausal breast cancer survivors

Physical activity	Number	Events	Overall mortality, HR (95% CI)	Events	Breast cancer mortality, HR (95% CI)	Events	Recurrence-free survival, HR (95% CI)
Pre- and postdiagnosis physical activity^a,b^	Predx/postdx	Predx/postdx		Predx/postdx		Predx/postdx	
No activity	693/818	76/115	1.00 (ref.)	43/64	1.00 (ref.)	115/157	1.00 (ref.)
Low activity	692/504	73/46	0.94 (0.74–1.19)	43/24	0.94 (0.68–1.29)	115/92	1.18 (0.98–1.43)
Sufficient activity	657/720	58/46	0.73 (0.57–0.93)	29/27	0.64 (0.46–0.89)	99/80	0.82 (0.68–0.99)
Prediagnosis physical activity^c^							
No activity	677	73	1.00 (ref.)	41	1.00 (ref.)	112	1.00 (ref.)
Low activity	676	73	1.07 (0.77–1.49)	43	1.18 (0.76–1.83)	112	1.05 (0.80–1.38)
Sufficient activity	647	58	0.97 (0.68–1.38)	29	0.90 (0.55–1.46)	97	1.04 (0.79–1.37)
Postdiagnosis physical activity^d^ in insufficiently active women prediagnosis							
No activity	662	91	1.00 (ref.)	53	1.00 (ref.)	121	1.00 (ref.)
Low activity	359	37	0.71 (0.48–1.06)	18	0.65 (0.37–1.16)	69	1.14 (0.84–1.55)
Sufficient activity	345	20	0.43 (0.26–0.72)	14	0.48 (0.25–0.91)	36	0.59 (0.40–0.86)
Postdiagnosis physical activity^d^ in sufficiently active women prediagnosis							
No activity	91	23	1.00 (ref.)	10	1.00 (ref.)	34	1.00 (ref.)
Low activity	37	9	0.38 (0.16–0.88)	6	0.69 (0.18–2.56)	20	0.75 (0.41–1.38)
Sufficient activity	20	26	0.57 (0.30–1.08)	13	0.59 (0.22–1.64)	44	0.65 (0.39–1.09)

^a^Leisure-time physical activity in MET-h/week was modelled as a time-dependent variable

^b^Analyses were adjusted for age at diagnosis, tumour size, nodal status, grade, ER/PR status, mode of detection, and menopausal hormone use at diagnosis, and were stratified by study centre and age at diagnosis in 5-year categories

^c^Analyses were adjusted for age at diagnosis, tumour size, nodal status, grade, ER/PR status, mode of detection, menopausal hormone use at diagnosis, chemotherapy, and hormone therapy, and were stratified by study centre and age at diagnosis in 5-year categories

^d^Analyses were adjusted for age at diagnosis, tumour size, nodal status, grade, ER/PR status, mode of detection, menopausal hormone use at diagnosis, and recurrences between diagnosis and follow-up, and were stratified by study centre and age at diagnosis in 5-year categories

## Discussion

In this analysis of 2042 postmenopausal long-term breast cancer survivors in the MARIE study, we observed that for women who were insufficiently physically active before breast cancer diagnosis but increased their physical activity postdiagnosis to recommended levels, there was a significant 50% reduction in overall mortality, a 46% reduction in breast cancer mortality, and a 42% improvement in recurrence-free survival compared to women who remained insufficiently active. There were also similar suggestions of improvements in prognosis with maintaining recommended levels of leisure-time physical activity pre- to postdiagnosis. That we do not see significantly decreased associations in this group could be attributed to the reference category, which includes both completely inactive women and insufficiently active women, thus potentially making it more difficult to detect associations and bias the results of the comparison groups to the null. Indeed, in the time-dependent analyses, where no activity is the reference group, sufficient activity is strongly and significantly associated with all three cancer outcomes. Our results suggest that achieving at least the recommended levels of moderate-intensity aerobic physical activity (7.5 MET-h/week), e.g. the equivalent of walking briskly (~ 5.6 km/h) for at least 150 min per week [13], after a breast cancer diagnosis, is beneficial to survival.

Our results indicating gains of maintaining or adopting an active lifestyle after breast cancer diagnosis are in line with other studies, where there was either a benefit to survival with increasing pre- to postdiagnosis physical activity [7], or harm with reducing pre- to postdiagnosis physical activity [5, 6]. Adherence to physical activity guidelines was also beneficial if women were able to adhere to guidelines post-treatment and 1-year post-treatment [8]. Specifically, our results demonstrate an improvement in prognosis with both adhering to guidelines (for those sufficiently active pre- and postdiagnosis) and increasing postdiagnosis leisure-time physical activity to recommended levels (for those insufficiently active prediagnosis). Results taken together than with those from other studies show that both increasing physical activity (> 9 MET-h/week before or after diagnosis [7]) increases survival and decreasing physical activity (decreasing > 3 MET-h/week pre- to postdiagnosis [6]) decreases survival [5, 6], independent of how physical activity change was categorized. Numerous mechanisms related to body fatness, sex hormones, growth factors, adipokines, immune function, and inflammation may be involved in mediating the impact of physical activity on survival [2, 3]. For example, results from a meta-analysis of five randomized controlled trials of postmenopausal breast cancer survivors showed that exercise after cancer therapy reduced levels of serum insulin growth factors and binding proteins [21].

In other studies examining pre- to postdiagnosis physical activity with breast cancer survival, postdiagnosis physical activity was assessed within 3 years after diagnosis [5–7]. Patients in our analysis had already survived a median of 5.8 years from breast cancer diagnosis, which restricts our results to long-term survivors of breast cancer. Women in our analysis, who had survived until and completed the follow-up questionnaire, were more likely to have exercised prediagnosis (median prediagnosis energy expenditure from leisure-time activities was 3.4 MET-h/week) compared to women who were alive but elected not to participate in the follow-up (1.0 MET-h/week) and women who died before the follow-up (median 0.2 MET-h/week). Therefore, our results may reflect a healthier subset of women. Also, pre- and postdiagnosis leisure-time physical activity was higher in women enrolled in the US studies [6, 7] compared to our own. We observe that adherence to the recommendations conferred an advantage to patients for improved prognosis compared to not adhering to the recommendations. That we see better prognosis for women who increased their leisure-time physical activity postdiagnosis to the World Health Organization’s physical activity guideline recommendations [17] compared to those who did not is encouraging for women who failed to adhere to the guidelines prediagnosis.

Even though a large proportion of women in our study were able to meet the World Health Organization’s and Germany’s national recommendations for physical activity, large randomized controlled trials of different domains of physical activity conducted at differing intensities and time points would be required to improve our knowledge about the complex relationship between pre- and postdiagnosis physical activity in breast cancer survivors to increase survival. Our results suggest that following current physical activity recommendations may be beneficial for prognosis following breast cancer diagnosis.

There are several strengths to our study including the large sample size. This is the first study outside the USA to evaluate the changes in physical activity using MET-h/week rather than on a 10-point ordinal scale [5] and evaluate the association between pre- to postdiagnosis physical activity based on levels that are relevant for public health and prognosis in postmenopausal breast cancer patients. Well-known prognostic factors [22, 23] including tumour size, nodal status, tumour grade, ER/PR tumour status, mode of detection, menopausal hormone use, tumour recurrences, and cancer therapies in addition to a wide range of demographic and lifestyle factors, which could have confounded and modified our associations of interest, were carefully and comprehensively accounted for in the analyses. We also examined the effect measure modification by several relevant factors. Postdiagnosis physical activity was ascertained at the follow-up and is likely to reflect long-term changes in behaviour after breast cancer diagnosis, surgery, and treatment. In addition to the overall and breast cancer mortality, we have also assessed recurrence-free survival, which includes non-death events that are predictors of death and a marker for survival [18]. Our follow-up time of 11.6 years after breast cancer diagnosis is also the longest of any study evaluating pre- to postdiagnosis physical activity and prognosis, allowing us to examine the long-term impact of physical activity as well as the changes in physical activity on cancer outcomes.

There are also some limitations to consider when interpreting our results. Physical activity was self-reported as in all other studies on this topic. Prediagnosis physical activity was assessed retrospectively after diagnosis, and postdiagnosis physical activity from 3 months after diagnosis to the follow-up was collected at the follow-up, creating a potential for recall bias, which could result in under- and overestimation of physical activity. This type of misclassification is likely to have been non-differential and would give attenuated associations with prognosis [24]. Another point to consider that was extensively described earlier is that in our analysis, the insufficiently active group includes a mix of women who are completely inactive (no leisure-time physical activities) and insufficiently active. Previous studies have demonstrated that as little as one to two sessions of weekly exercise associate with significant survival advantages in cancer survivors in comparison with those who do nothing [25, 26]. Therefore, because these women are also included in our reference group, risk estimates for the comparison groups are likely to be attenuated or less significant than if we had only included women who were completely inactive. Findings from time-dependent models help to clarify these associations.

There is also the possibility of reverse causation in women who could not exercise because they were too sick (decreasingly active) and women who wanted to change their behaviours and were able to exercise due to being less sick (increasingly active). Proportionally, tumour size, tumour grade, and hormone receptor status were similar between women with decreased activity and women with increased activity. However, there were relatively more women with more than three positive lymph nodes who had increased activity than those who had decreased activity, meaning that those who had a more advanced disease were actually women who we may have expected to become insufficiently active postdiagnosis. Reverse causation could also stem from comorbidities, which we had tested. We have accounted for possible confounding by including the prognostic factors tumour size, nodal status, tumour grade, and hormone receptor status in our models. We have also examined physical activity based on leisure-time physical activity only and not total physical activity including commuting, household, or occupational activities. Previous studies on physical activity and survival after breast cancer diagnosis [6] including one from the MARIE study [14] have shown the strongest associations between leisure-time physical activity and survival.

## Conclusions

We have observed an improved overall breast cancer prognosis amongst postmenopausal long-term breast cancer survivors who engaged in at least 150 min per week of moderate-intensity physical activity postdiagnosis regardless of physical activity level prediagnosis. Our results, in combination with other studies, suggest that physical activity is important in improving survival following breast cancer diagnosis and should be encouraged both pre- and postdiagnosis and perhaps more crucially so in women who were insufficiently active prediagnosis.

## Data Availability

The datasets generated and/or analysed during the current study are not publicly available due to individual patient privacy but are available from the corresponding author on reasonable request.

## Abbreviations

BMI: Body mass index
CI: Confidence interval
ER: Oestrogen receptor
HER2: Human epidermal growth factor receptor 2
HR: Hazard ratio
IQR: Interquartile range
MET-h/week: Metabolic equivalent task-hours per week
PR: Progesterone receptor

## References

[1] World Cancer Research Fund International/American Institute for Cancer Research Continuous Update Project Expert Report 2018. Diet, nutrition, physical activity, and breast cancer survivors. Available at dietandcancerreport.org.

[2] Picon-Ruiz M, Morata-Tarifa C, Valle-Goffin JJ, Friedman ER, Slingerland JM (2017). Obesity and adverse breast cancer risk and outcome: mechanistic insights and strategies for intervention. CA Cancer J Clin.

[3] Chlebowski RT, Aiello E, McTiernan A (2002). Weight loss in breast cancer patient management. J Clin Oncol.

[4] Mason C, Alfano CM, Smith AW, Wang CY, Neuhouser ML, Duggan C, Bernstein L, Baumgartner KB, Baumgartner RN, Ballard-Barbash R (2013). Long-term physical activity trends in breast cancer survivors. Cancer Epidemiol Biomark Prev.

[5] Borch KB, Braaten T, Lund E, Weiderpass E (2015). Physical activity before and after breast cancer diagnosis and survival - the Norwegian women and cancer cohort study. BMC Cancer.

[6] Irwin ML, Smith AW, McTiernan A, Ballard-Barbash R, Cronin K, Gilliland FD, Baumgartner RN, Baumgartner KB, Bernstein L (2008). Influence of pre- and postdiagnosis physical activity on mortality in breast cancer survivors: the health, eating, activity, and lifestyle study. J Clin Oncol.

[7] Irwin ML, McTiernan A, Manson JE, Thomson CA, Sternfeld B, Stefanick ML, Wactawski-Wende J, Craft L, Lane D, Martin LW (2011). Physical activity and survival in postmenopausal women with breast cancer: results from the women’s health initiative. Cancer Prev Res (Phila).

[8] Bertram LA, Stefanick ML, Saquib N, Natarajan L, Patterson RE, Bardwell W, Flatt SW, Newman VA, Rock CL, Thomson CA (2011). Physical activity, additional breast cancer events, and mortality among early-stage breast cancer survivors: findings from the WHEL study. Cancer Causes Control.

[9] Flesch-Janys D, Slanger T, Mutschelknauss E, Kropp S, Obi N, Vettorazzi E, Braendle W, Bastert G, Hentschel S, Berger J (2008). Risk of different histological types of postmenopausal breast cancer by type and regimen of menopausal hormone therapy. Int J Cancer.

[10] Jaskulski S, Jung AY, Behrens S, Johnson T, Kaaks R, Thone K, Flesch-Janys D, Sookthai D, Chang-Claude J (2018). Circulating enterolactone concentrations and prognosis of postmenopausal breast cancer: assessment of mediation by inflammatory markers. Int J Cancer.

[11] Pols MA, Peeters PH, Ocke MC, Bueno-de-Mesquita HB, Slimani N, Kemper HC, Collette HJ (1997). Relative validity and repeatability of a new questionnaire on physical activity. Prev Med.

[12] Steindorf K, Schmidt M, Kropp S, Chang-Claude J (2003). Case-control study of physical activity and breast cancer risk among premenopausal women in Germany. Am J Epidemiol.

[13] Ainsworth BE, Haskell WL, Whitt MC, Irwin ML, Swartz AM, Strath SJ, O’Brien WL, Bassett DR, Schmitz KH, Emplaincourt PO (2000). Compendium of physical activities: an update of activity codes and MET intensities. Med Sci Sports Exerc.

[14] Schmidt ME, Chang-Claude J, Vrieling A, Seibold P, Heinz J, Obi N, Flesch-Janys D, Steindorf K (2013). Association of pre-diagnosis physical activity with recurrence and mortality among women with breast cancer. Int J Cancer.

[15] U.S. Department of Health and Human Services (2018). Physical activity guidelines for Americans.

[16] Pfeifer K, Rutten A. National Recommendations for Physical Activity and Physical Activity Promotion. Gesundheitswesen. 2017;79(S 01):S2-S3. https://www.ncbi.nlm.nih.gov/pubmed/28399579. 10.1055/s-0042-123346 28399579

[17] World Health Organization (2010). Global recommendations on physical activity for health.

[18] Hudis CA, Barlow WE, Costantino JP, Gray RJ, Pritchard KI, Chapman JA, Sparano JA, Hunsberger S, Enos RA, Gelber RD (2007). Proposal for standardized definitions for efficacy end points in adjuvant breast cancer trials: the STEEP system. J Clin Oncol.

[19] Grambsch P, Louis TA, Bostick RM, Grandits GA, Fosdick L, Darif M, Potter JD (1994). Statistical analysis of proliferative index data in clinical trials. Stat Med.

[20] Fisher LD, Lin DY (1999). Time-dependent covariates in the Cox proportional-hazards regression model. Annu Rev Public Health.

[21] Meneses-Echavez JF, Jimenez EG, Rio-Valle JS, Correa-Bautista JE, Izquierdo M, Ramirez-Velez R (2016). The insulin-like growth factor system is modulated by exercise in breast cancer survivors: a systematic review and meta-analysis. BMC Cancer.

[22] Soerjomataram I, Louwman MW, Ribot JG, Roukema JA, Coebergh JW (2008). An overview of prognostic factors for long-term survivors of breast cancer. Breast Cancer Res Treat.

[23] Galea MH, Blamey RW, Elston CE, Ellis IO (1992). The Nottingham Prognostic Index in primary breast cancer. Breast Cancer Res Treat.

[24] Greenland S, Lash TL, Rothman KJ, Greenland S, Lash TL (2008). Bias analysis. Modern epidemiology.

[25] O’Donovan G, Lee IM, Hamer M, Stamatakis E (2017). Association of “weekend warrior” and other leisure time physical activity patterns with risks for all-cause, cardiovascular disease, and cancer mortality. JAMA Intern Med.

[26] Cannioto RA, Dighe S, Mahoney MC, Moysich KB, Sen A, Hulme K, McCann SE, Ambrosone CB (2019). Habitual recreational physical activity is associated with significantly improved survival in cancer patients: evidence from the Roswell Park Data Bank and BioRepository. Cancer Causes Control.

